# Surveillance of SARS‐CoV‐2 infection among frontline health care workers in Wuhan during COVID‐19 outbreak

**DOI:** 10.1002/iid3.340

**Published:** 2020-08-20

**Authors:** Xin Tong, Mingzhe Ning, Rui Huang, Bei Jia, Xiaomin Yan, Yali Xiong, Weihua Wu, Jiacheng Liu, Yuxin Chen, Chao Wu

**Affiliations:** ^1^ Department of Infectious Diseases, Nanjing Drum Tower Hospital Nanjing University Medical School Nanjing Jiangsu China; ^2^ Department of Laboratory Medicine, Nanjing Drum Tower Hospital Nanjing University Medical School Nanjing Jiangsu China

## Abstract

**Introduction:**

As an emerging infectious disease, coronavirus disease 2019 (COVID‐19) has rapidly spread throughout worldwide. Health care workers (HCWs) on frontline directly participated in the diagnosis, treatment, and care of COVID‐19 patients are at high risk of getting infected with the highly infectious severe acute respiratory syndrome coronavirus 2 (SARS‐CoV‐2), the novel coronavirus that causes COVID‐19. In Nanjing Drum Tower Hospital, a total of 222 medical staff went to Wuhan city for support. In this study, we aimed to determine any nosocomial infection among our cohort of HCWs who worked in Wuhan.

**Methods:**

Throat swab samples were obtained for RNA testing on day 1 and 14 of their quarantine upon their return to Nanjing. Radiological assessments were performed by chest computed tomography (CT) on day 14 of their quarantine. The blood was collected from 191 HCWs between May 12 and May 15. Anti‐SARS‐CoV‐2 immunoglobulin M (IgM) and IgG antibody responses were determined by a chemiluminescence immunoassay.

**Results:**

All the throat swab specimens were found negative for SARS‐CoV‐2. The radiological analysis revealed that there was no typical chest CT scan of COVID‐19 among 222 HCWs. Consistently, anti‐SARS‐CoV‐2 IgM or IgG was also found to be negative among 191 HCWs.

**Conclusions:**

There was no nosocomial infection of SARS‐CoV‐2 among our cohort of the frontline HCWs, suggesting that zero occupational infection is an achievable goal with appropriate training, strict compliance, and psychological support for the frontline HCWs.

## Graphical abstract

Although coronavirus disease 2019 is a highly communicable disease, that zero occupational infection of severe acute respiratory syndrome coronavirus 2 is an achievable goal with appropriate training, strict compliance, and psychological support for frontline health care workers.

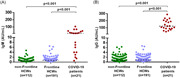

Severe acute respiratory syndrome coronavirus 2 (SARS‐CoV‐2) is an emerging infectious disease, first described in Wuhan, China, has rapidly spread throughout worldwide.^1^ Because of efficient transmission of SARS‐CoV‐2, health care workers (HCWs) on frontline directly involved in the diagnosis and treatment of coronavirus disease 2019 (COVID‐19) patients are at high risk of getting an infection of SARS‐CoV‐2.^2^ The ever‐increasing number of COVID‐19 cases, overwhelming workload, the depletion of personal protection equipment (PPE), physical fatigue, and psychological stress during the early outbreak has resulted in at least 22 073 cases of COVID‐19 among HCWs.^3^ A study from China Center for Disease Control and Prevention (CDC) showed that as of 17 February 2020, 3.8% confirmed COVID‐19 cases were among HCWs.^4^ A report from Italy revealed 11% of COVID‐19 cases were HCWs.^5^ All the evidence suggested a high risk of occupational infection of SARS‐CoV‐2.

In China, a large number of HCWs from various provinces in China went to Wuhan city for support. In Nanjing Drum Tower Hospital, a tertiary hospital in Nanjing city of China, a total of 222 medical staff, including 63 doctors and 159 nurses stayed in three medical centers in Wuhan City, respectively. Four medical staffs worked in First people's Hospital of Jiangxia District from 26 January to 17 March, 56 medical staffs served in Tongji Hospital from 9 February to 31 March while 162 medical professionals first worked at Wuhan No.1 Hospital and later transferred to Hubei General Hospital from 13 February to 31 March. In this study, we aimed to determine any nosocomial infection among our cohort of HCWs who worked in Wuhan.

Prior to their departure from Nanjing, HCWs received a group training of SARS‐CoV‐2, including the transmission route, the diagnosis, the clinical manifestation, and treatment guidance of COVID‐19. Upon their arrival to Wuhan, they received an infection prevention and control training program held by the local hospitals, including detailed procedures of donning, doffing, and disposal of PPE as well as hand hygiene. The PPE includes N95 respirator, coverall gown, goggle/face shield, and glove. During their stay in Wuhan, these HCWs stayed in the contaminated area every 4 h/d, including performing aerosol‐generating procedures, collecting or handling specimens, providing care for COVID‐19 patients, and sharing conversations with COVID‐19 patient within a one‐meter reach. No HCWs reported COVID‐19 clinical symptoms during their stay in Wuhan.

To further identify any possible infection of SARS‐CoV‐2, the seroprevalence, nucleic acid assay, and chest computed tomography (CT) of SARS‐CoV‐2 among 222 HCWs were performed when they were back to Nanjing. Upon their return to Nanjing, they started a 14‐day quarantine. Throat swab samples were obtained for RNA testing on day 1 and 14 of their quarantine. Viral RNA was tested using real‐time reverse transcriptional polymerase chain reaction kit (BGI Genomics, Beijing, China) as recommended by the Chinese CDC following WHO guidelines.^1^ Radiological assessments were performed by chest CT on day 14 of their quarantine. The blood was collected from 191 HCWs between 12 May and 15 May. Anti‐SARS‐CoV‐2 immunoglobulin M (IgM) and IgG antibody responses were determined by a chemiluminescence immunoassay‐based test developed by YHLO Biotech Co, Ltd, (Shenzhen, China). This study was approved by the ethics committee of our hospital. Written informed consent was waived by the Ethics Commission due to a public health outbreak investigation.

The mean age of these HCWs was 32 years (range: 24‐58) and 57 (25.67%) were male. They worked 4 to 6 hour shifts for an average of 5.6 days a week. All the throat swabs collected on day 3 and 14 of the quarantine were found negative for SARS‐CoV‐2. The radiological analysis revealed that there was no typical chest CT scan of COVID‐19 among 222 HCWs (Table 1).^6^ Consistently, anti‐SARS‐CoV‐2 IgM or IgG were also found to be negative among 191 HCWs, negative response to SARS‐CoV‐2 was detected in the 112 control HCWs with no history of exposure to COVID‐19 patients. As positive controls, 21 serum samples from COVID‐19 patients had high titers of either SARS‐CoV‐2 IgM or IgG (Figure 1). Based on these results, there was no nosocomial infection of SARS‐CoV‐2 in our cohort.

**Table 1:** Demographic and baseline characteristics of frontline HCWs

Characteristics	Total (n = 222)	Doctors (n = 63)	Nurses (n = 159)
Male	57	42	19
Female	165	21	140
Average age, y	32	37	30
SARS‐CoV‐2 viral RNA	Negative	Negative	Negative
CT	Normal	Normal	Normal
IgM	Negative	Negative	Negative
IgG	Negative	Negative	Negative
Working hours			
Shifts, h/shift	4	4	4
Work, d/wk	5.6	5.6	5.6

Abbreviations: CT, computed tomography; HCW, health care worker; IgM, immunoglobulin M; SARS‐CoV‐2, severe acute respiratory syndrome coronavirus 2.

**Figure 1:**
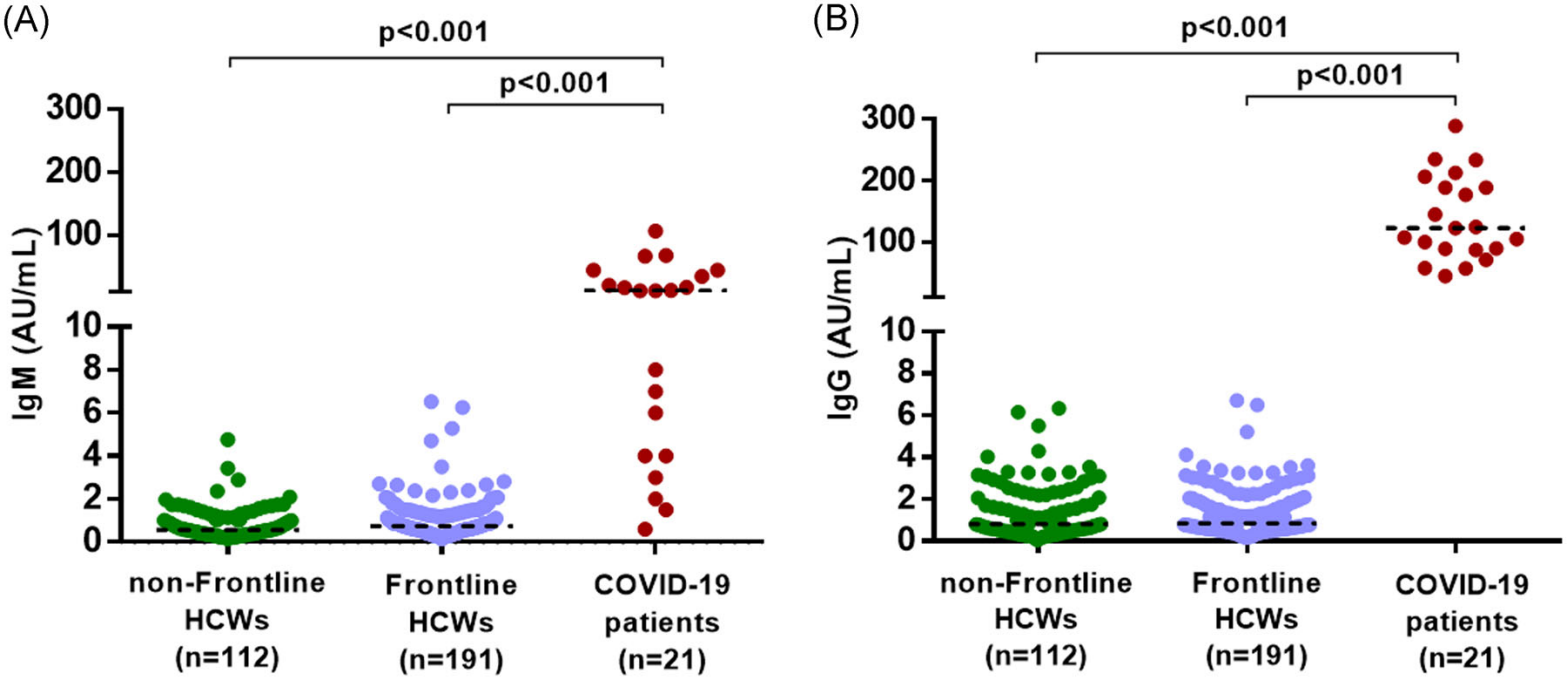
Serological response to SARS‐CoV‐2. The level of SARS‐CoV‐2 specific IgM and IgG antibodies in serum samples of the frontline HCWs in Wuhan were quantified by chemiluminescence immunoassay (n = 191). Serum samples from control HCWs without COVID‐19 exposure were served as a negative control (n = 112). Serum samples from COVID‐19 patients were used as positive controls (n = 21). Reference specified by the manufacturer (<10 AU/mL). COVID‐19, coronavirus disease 2019; HCW, health care worker; IgM, immunoglobulin M; SARS‐CoV‐2, severe acute respiratory syndrome coronavirus 2

Our results revealed that zero occupational infection is an achievable goal among our frontline HCWs. This could be attributed by several reasons. First, comprehensive site training and an electronic reminder of infection prevention and control programs were carried out. Second, our established infection prevention and control program was strictly adhered and constantly surveilled. Third, our HCWs were given substantial psychological and nutritional support during their stay in Wuhan.

Our study also has several limitations. First, during their stay in Wuhan, the throat swab samples were not collected routinely to determine any possible viral infection of SARS‐CoV‐2 among frontline HCWs. Second, blood samples were only collected at a one‐time point when they were back in Nanjing for the anti‐SARS‐CoV‐2 IgM and IgG testing.

To conclude, although COVID‐19 is a highly communicable disease, zero occupational infection of SARS‐CoV‐2 is an achievable goal with appropriate training, strict compliance, and psychological support for frontline HCWs.

## CONFLICT OF INTERESTS

The authors declare that there are no conflict of interests.

## AUTHOR CONTRIBUTIONS

XT, YC, and CW contributed to the study concept and design, XT, YC, RH, and BJ contributed to the acquisition of data, analysis and interpretation of data, and critical revision of the manuscript. MN, JL, and WW contributed to investigation and methodology. XY and YX contributed to resources and software.

## Data Availability

The data that support the findings of this study are available from the corresponding author upon reasonable request.
